# Insect-specific RNA viruses detection in Field-Caught *Aedes aegypti* mosquitoes from Argentina using NGS technology

**DOI:** 10.1371/journal.pntd.0012792

**Published:** 2025-01-10

**Authors:** Lucas Ripoll, Javier Iserte, Carolina Susana Cerrudo, Damian Presti, José Humberto Serrat, Ramiro Poma, Federico Alejandro Javier Mangione, Gabriela Analía Micheloud, Verónica Viviana Gioria, Clara Inés Berrón, M. Paola Zago, Cristina Borio, Marcos Bilen

**Affiliations:** 1 Laboratorio de Ingeniería Genética y Biología Celular y Molecular—Área de virus de insectos, Departamento de Ciencia y Tecnología, Universidad Nacional de Quilmes, Quilmes, Buenos Aires, Argentina; 2 Laboratorio de Bioinformática Estructural, Fundación Instituto Leloir, Ciudad de Buenos Aires, Buenos Aires, Argentina; 3 Programa de Zoonosis, Dirección General de Coordinación Epidemiológica-Ministerio de Salud Pública de Salta, Salta, Salta, Argentina; 4 Unidad de Conocimiento Traslacional Hospitalaria, Hospital Público Materno Infantil de Salta (UCT-HPMI)-CONICET, Salta, Salta, Argentina; 5 Laboratorio de Virología, Facultad de Bioquímica y Ciencias Biológicas, Universidad Nacional del Litoral, Santa Fe, Santa Fe, Argentina; University of California Davis, UNITED STATES OF AMERICA

## Abstract

Mosquitoes are the primary vectors of arthropod-borne pathogens. *Aedes aegypti* is one of the most widespread mosquito species worldwide, responsible for transmitting diseases such as Dengue, Zika, and Chikungunya, among other medically significant viruses. Characterizing the array of viruses circulating in mosquitoes, particularly in *Aedes aegypti*, is a crucial tool for detecting and developing novel strategies to prevent arbovirus outbreaks. In this study, we address the implementation of a sequencing and analysis pipeline based on the Oxford Nanopore Technologies MinION Mk1b system, for arboviral detection in field-caught mosquitoes from Argentina. Full genome of Humaita Tubiacanga Virus (HTV), Phasi Charoen-like Phasivirus (PCLV), Aedes aegypti totivirus (AaeTV) has been sequenced in three distinct regions of Argentina comprising Buenos Aires province, Santa Fe province and the northern province of Salta. Viral sequences enriched by SISPA and coupled with Nanopore sequencing can be a useful tool for viral surveillance, not only for detecting viruses that have a high impact on human and animal health, but also for detecting insect-specific viruses that could promote the transmission of arboviruses.

## Author summary

Mosquitoes are well-known vectors of diseases that impact both human and animal health. Among them, the *Aedes aegypti* mosquito, distributed globally, is particularly notable for transmitting viruses such as Dengue, Zika, and Chikungunya. Developing robust surveillance methods and gaining a comprehensive understanding of the viruses these mosquitoes harbor is essential for preventing disease outbreaks. In this study, we employed Oxford Nanopore Technologies’ sequencing platform to detect viruses in mosquitoes collected from various regions of Argentina. Our findings revealed the presence of several viruses, including mosquito-specific viruses, across different parts of the country. This approach not only helps identify viruses that pose a direct threat to humans and animals but also those that exclusively affect mosquitoes, which may indirectly influence disease transmission dynamics. Our research provides a valuable surveillance tool that can be used not only to help prevent outbreaks but also to gather critical information during ongoing outbreaks.

## 1. Introduction

The first hypothesis of arthropod-borne disease transmission was formulated more than hundred years ago [1]. Since that time, numerous diseases with high impact in the human health population have been found to be associated with mosquito-borne transmission [2–3]. The widespread of mosquitoes around the world, particularly the *Aedes spp*., modulated by anthropogenic activity and also driven by climate change makes the mosquito’s borne viruses a major public health threat [4–5].

*Aedes aegypti* has its roots in a sylvan ancestor from sub-Saharan Africa, which made its way to West Africa in the late eighth century. It is likely that the mosquito was brought to the New World through the African slave trade, occurring between the fifteenth and seventeenth centuries [6]. This species is the primary vector of Dengue (DENV Serotypes 1–4), Zika (ZIKV), Chikungunya (CHKV) and Yellow Fever (YFV) [7]. Moreover, it has been established that mosquitoes serve as hosts for a broad spectrum of arboviruses and Insect-specific viruses (ISVs), specifically known as mosquito-specific viruses (MSVs). MSVs infect either mosquitoes or cells derived from mosquitoes. The identification of MSVs has significantly broadened our comprehension of viral diversity and has shed light on the intricate interactions between these viruses when they co-infect mosquitoes alongside arthropod-borne viruses (arboviruses) [8]. The presence of MSVs can either suppress or enhance the mosquito’s infection by arboviruses of significant human health importance [9–10]. For example, the presence of Phasi Charoen-like phasivirus (PCLV) and Humaita-Tubiacanga virus (HTV) in *Aedes aegypti* was recently shown to have a twofold increase in the chances of the mosquito being infected by Dengue virus [11].

The extensive adoption of high-throughput sequencing (HTS) has significantly influenced viromics studies. The widespread utilization of cost-effective and easily accessible massive parallel sequencing platforms has led to a substantial increase in viromic analyses in mosquito samples worldwide, particularly focusing on RNA viruses [12–13]. Regardless of the chosen sequencing platform for virome sequencing, these methods rely on the use of random priming and the amplification of viral sequences in combination with high-throughput sequencing tools [13]. These approaches serve as valuable tools for characterizing the mosquito virome and have led to the discovery of a high diversity of MSVs [14–18].

One of the widely adopted methods for enriching the viral RNA genome in a few steps is the Sequence-independent single primer amplification (SISPA) [19]. This technique involves the use of random oligos anchored to a barcode during the initial retrotranscription of the entire RNA from a sample. Subsequently, a second-strand synthesis is performed using the same primer employed in the first step. The result of these two steps is an unknown double-stranded DNA (dsDNA) sequence flanked by a barcode sequence. The third step encompasses the complete amplification of this product using the barcode primer. Subsequent sequencing of this product and further bioinformatic analysis contribute to viral detection or virome characterization [20–22].

Furthermore, SISPA is widely used couple to third generation sequencing platforms. The combination of both systems was employed for the precise identification of etiological agents, as well as for epidemiological monitoring and virome characterization in several types of samples [23–26].

This study aims to implement a SISPA based method coupled to Nanopore sequencing to monitor the distribution of mosquito-borne viruses, particularly *Aedes aegypti*, in Argentina. The main purpose is to provide a surveillance tool for the detection of circulating viruses specific to the vector and also viruses that have a significant impact on both human and animal health, as well as enabling the early detection of viral emergencies.

## 2. Material and Methods

### 2.1. Mosquito sample capture

**Fig 1:**
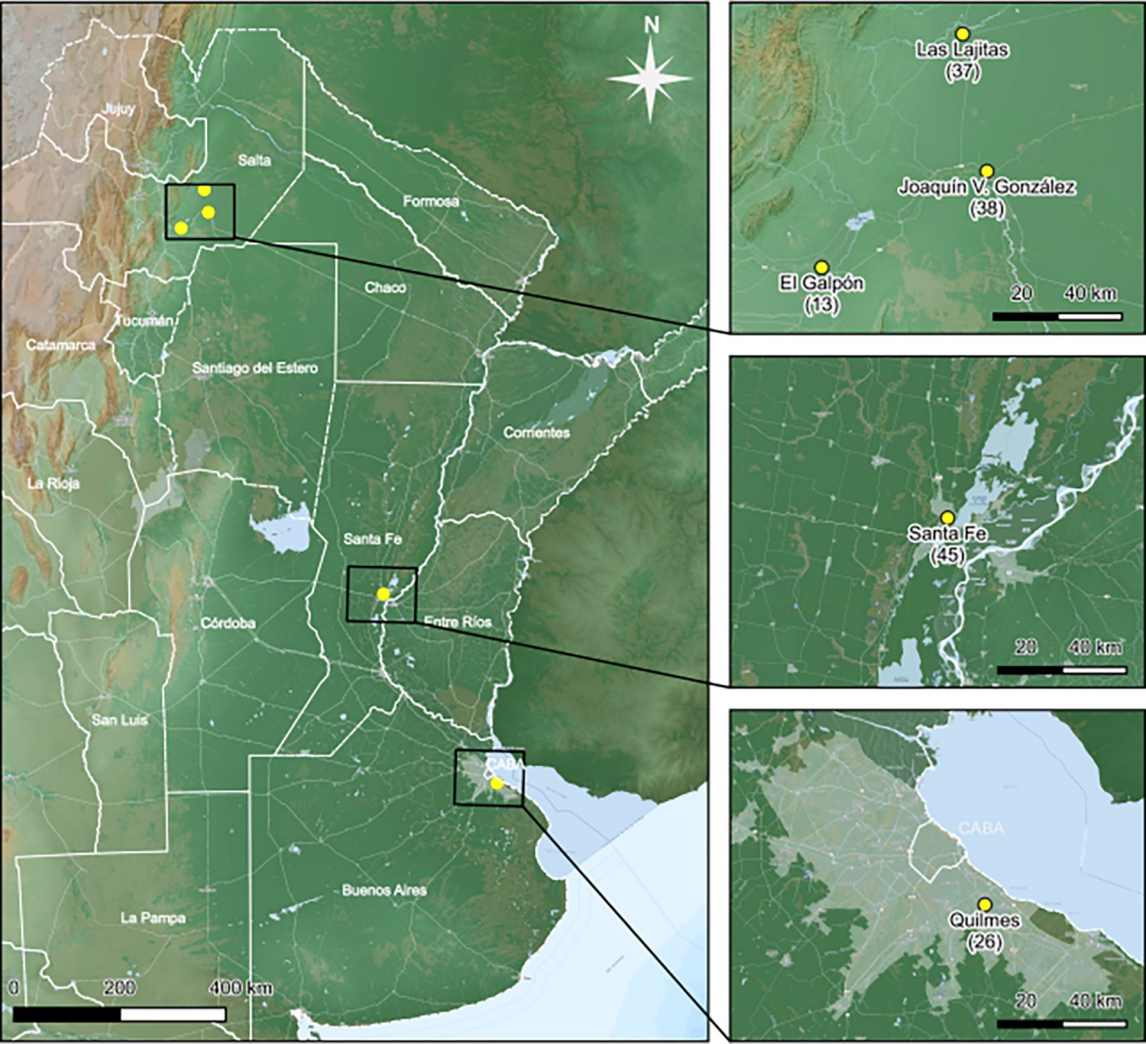
Map of sample collection sites: Distribution of sample collection sites in three provinces of Argentina, Buenos Aires, Santa fe and Salta. Each collection site shows the number of mosquitoes processed in each sample. Basemap data were sourced from Argenmap by the Instituto Geográfico Nacional de Argentina (IGN, https://www.ign.gob.ar) and OpenStreetMap (https://www.openstreetmap.org). Both datasets are provided under the Open Database License (ODbL, https://opendatacommons.org/licenses/odbl/1-0/).

Adult *Aedes aegypti* mosquitoes were captured and morphologically classified between December 2022 and May 2023 (Table 1) from three distinct regions across Argentina (Fig 1). These samples were collected during the Dengue and Chikungunya outbreak that occurred during that period. The first set of samples was acquired from the Buenos Aires metropolitan area, specifically in the city of Quilmes. The second set of samples was collected in the Northwest province of Salta during May 2023, encompassing the cities of Las Lajitas, Galpon, and Joaquín V. González. In both regions, samples were captured using a battery-powered aspirator. The specimens were stored in RNA stabilizer (PB-L, Argentina) for transportation.

The third collection site was the city of Santa Fe in the province of Santa Fe. Here *Aedes aegypti* eggs were collected using ovitraps and reared to the adult stage. In all cases, samples were grouped into pools and preserved in RNA stabilizer (PB-L, Argentina) for transportation. Upon arrival at the lab, RNA stabilizer was removed and samples were stored at -80° C degrees.

**Table 1:** Field Mosquito Samples collected: Number of mosquitoes processed per sample and corresponding site of collection.

Sample number	Amount	City—Province	Coordinates	Collection date
1	26	Quilmes—Buenos Aires	*-34*.*764300*, *-58*.*273152*	*December 2022*
2	45	City of Santa Fe—Santa Fe	*-31*.*610806*, *-60*.*701455*	*December 2022*
3	13	Galpon—Salta	*-25*.*378760*, *-64*.*639162*	*May 2023*
4	37	Las Lajitas—Salta	*-24*.*726449*, *-64*.*196061*	*May 2023*
5	38	Joaquín V. González—Salta	*-25*.*113005*, *-64*.*124737*	*May 2023*

### 2.2. Library preparation

#### 2.2.1. Sample homogenization

The mosquito samples were mixed with 300 μL of SM buffer (50 mM Tris, 10 mM MgSO4, 0.1 M NaCl, pH 7.5) and homogenized using a sterile polypropylene pestle. Subsequently, the pools were centrifuged at 12,000 xg for 10 minutes. The supernatant was then recovered to separate it from mosquito debris and other materials.

#### 2.2.2. RNase and DNase treatment

The supernatant was treated with 1 U of DNase I (PB-L), 10 U of RNase A (PB-L), and 10X DNase buffer (PB-L) were added to 133 μL of the supernatants to a final volume of 150 μL, followed by incubation at 37°C for 1 h, in order to digest host genomic DNA and the non-enveloped and free RNA.

#### 2.2.3. RNA extraction and purification

After removing free nucleic acid and host DNA, the total remaining enveloped and encapsulated RNA was extracted and purified using Bio-Zol (PB-L) following the manufacturer’s instructions. The total RNA was then resuspended in 15 μL of nuclease-free water.

#### 2.2.4. First strand synthesis

The total RNA obtained after the prior purification step was reverse transcribed using Nonamer-anchored barcode primers (Table 2) and M-MLV reverse transcriptase (PB-L). For denaturation, 1 μL of 100 μM primers was added separately to 5 μL of RNA extraction and incubated at 65°C for 5 min, followed by placement on ice for 5 min. Then was added 40 U of RNase inhibitor (PB-L), 200 U of M-MLV, 1 μL of 10 mM dNTPs, 4 μL of 5X first-strand buffer, and nuclease-free water was added to reach a final volume of 20 μL. The reaction was incubated 10 min at 25°C followed by 60 min at 42°C.

**Table 2:** Primer table used for first and second strand synthesis and SISPA.

Primer type	Primer	Sequence (5’->3’)
Random anchored barcode	Nonamer-anchored BC01	AAGAAAGTTGTCGGTGTCTTTGTGNNNNNNNNN
	Nonamer-anchored BC02	TCGATTCCGTTTGTAGTCGTCTGTNNNNNNNNN
	Nonamer-anchored BC03	GAGTCTTGTGTCCCAGTTACCAGGNNNNNNNNN
	Nonamer-anchored BC04	TTCGGATTCTATCGTGTTTCCCTANNNNNNNNN
	Nonamer-anchored BC05	CTTGTCCAGGGTTTGTGTAACCTTNNNNNNNNN
Phosphorylated barcode primer	BC01	AAGAAAGTTGTCGGTGTCTTTGTG
	BC02	TCGATTCCGTTTGTAGTCGTCTGT
	BC03	GAGTCTTGTGTCCCAGTTACCAGG
	BC04	TTCGGATTCTATCGTGTTTCCCTA
	BC05	CTTGTCCAGGGTTTGTGTAACCTT

#### 2.2.5. Second strand synthesis

After the first strand synthesis, 1 μL of RNase H (New England Biolabs—NEB) was added to the retrotranscription product to eliminate any remaining RNA hybridizing to the cDNA, followed by an incubation at 37° C for 60 minutes. The second strand synthesis utilized 5 U of Klenow Exo (5’-3’-) (NEB), 2 μL of 100 μM Nonamer-anchored barcode (Table 2), 1 μL of 10 mM dNTPs, 2 μL of 10X Buffer NEB 2 (NEB), 8 μL of cDNA from the previous step, and nuclease-free water, to reach a final volume of 20 μL. The reaction included incubation at 4° C for 10 minutes, followed by a second step at 25° C for 10 minutes, a third step at 37° C for 60 minutes, and a final step at 75° C for 10 minutes. Subsequently, to eliminate ssDNA and free phosphate, the reaction was incubated with 10 U of Thermolabile Exonuclease I (NEB), 1 U of Thermolabile Shrimp Alkaline Phosphatase (NEB), in 1X CutSmart Buffer (NEB), in afinal volume of 50 μL. The reaction was incubated at 37° C for 60 minutes, followed by a heat denaturation at 75° C for 10 minutes.

#### 2.2.6. Sequence-independent single primer amplification (SISPA)

The reaction was performed adding 10 μL of dscDNA from the previous reaction, 2.5 μL of 10 mM dNTPs, 1.5 μL of 50 mM MgCl2, 2.5 μL of 10 μM of the corresponding phosphorylated barcode primer for each sample (Table 2), 2.5 U of Taq Pegasus (PB-L), 5 μL 10X Taq Pegasus Buffer, and nuclease-free water, reaching a final volume of 50 μL. The reaction involved an initial denaturation step at 92° C for 5 minutes, followed by 30 cycles of 92° C for 15 seconds, 55° C for 15 seconds, and 72° C for 2 minutes, with a final extension step at 72° C for 5 minutes. Gel electrophoresis on a 2% agarose gel was used to visualize the effective amplification of each sample and estimate the average fragment size post amplification. After verification, all samples were subsequently purified using the Attractor System (PB-L). The DNA was eluted in 20 μL of nuclease-free water, and the purified products were quantified using the Qubit 3.0 with the Quant-iT dsDNA Broad-Range Assay Kit (Thermo Fisher Scientific).

### 2.3. Sequencing

Each sample of barcoded amplicons resulting from SISPA was pooled in equimolar quantities for sequencing adapter ligation. For the sequence adapter ligation 10 fmol of each sample were pooled and ligated to sequencing adapters using SQK-LSK109 kit from Oxford Nanopore Technologies (ONT). Adapter ligated libraries were loaded onto the SpotON flow-cell (R.9.4.1) and sequencing was performed using the MinION Mk1B system (ONT) and sequencing runs were performed for about 24 h using Min-KNOW software (ONT) with a cutoff of Q = 7 and reads ≧ 200 pb.

### 2.4. Bioinformatic pipeline

To obtain the genomic sequences of the viruses present in the different samples, an *ad hoc* bioinformatic pipeline, described below, was generated based on Python script. Raw Reads in Fast5 format were converted to POD5 format using POD5 Tools (v. 0.2.4). Basecalling and conversion to FastQ format was performed using Dorado (v. 0.3.4, https://github.com/nanoporetech/dorado; accessed in September 2023) with a quality cutoff of Q = 10. Basecalling model used was dna_r9.4.1_e8_sup@v3.6. The reads were trimmed and demultiplexed using Oxford Nanopore Technologies Guppy barcoder.

Identification of viral related reads was performed through many local BLASTN [27] searches. The first search was made against the complete non redundant virus database (nt_viruses). Then, against bacterial, and eukaryotic rRNA databases (16S_ribosomal_RNA, 18S_fungal_sequences, 28S_fungal_sequence, ITS_eukaryotic_sequences, LSU_eukaryote_rRNA, LSU_prokaryote_rRNA). Finally, against the human genome (GRCh38.p14) and *Aedes aegypti* genome assembly AaegL5.0. For date accession and full links to databases see S1 Table. In all the cases we use a cut-off e-value of 1e-5.

In order to avoid the inclusion of reads derived from endogenous viral elements (EVEs) from *Aedes aegypti*, we performed the following: The BLASTN hits matching the viral database were filtered using the results from the hits to the rRNA, *Aedes aegypti* genome assembly AaegL5.0 and human databases. When a viral hit matched any of the latter, the bit score value from BLASTN was used to make a decision. If the bit score of the viral hit is greater than the rRNA, *Aedes aegypti* AaegL5.0 or human hit the read is included; otherwise is discarded (S2 Table). The filtered reads were divided by organism, based on the species TaxID of the target (S3 Table). Viral hits were mapped against a reference using Minimap2 (v. 2.24) indicating to the software that the reads used in the alignment to a reference genome are noisy long reads from ONT (-x map-ont option). The other parameters of Minimap2 (v.2.24) were set by default [28]. BAM files were generated using Samtools (v. 1.17) [29]. Coverage at each position was computed using the Samtools (v. 1.17) coverage tool and then the histograms were generated with SigmaPlot (v. 11.0). Viral consensus sequences were obtained from the BAM files in FASTA format using Samtools (v. 1.17). Due to the possibility of collapsing viral quasispecies during reference alignment, the frequency of each base was evaluated, position by position, in each of the fully assembled viral segments. This was done with the aim of visualizing SNP frequency in each assembly.

### 2.5. Phylogeny inference

For each novel viral genome, phylogenetic inference was performed using previously established gene or protein sequences for identification (or taxonomic assignment) according to the literature. In all cases, a search for similar sequences was conducted using the GenBank virus database and BLASTPor BLASTN, as appropriate. Clustal Omega was employed to generate the alignments [30] and sequence identity percentage matrices were recovered to perform comparative analyses (Tabla Sup_Identidad). Phylogenetic inference was carried out using the Maximum Likelihood (ML) method, utilizing IQ-TREE 2.1.2 [31] and selecting “ModelFinder + tree reconstruction + ultrafast bootstrap (1000 replicates)” for the analysis. Finally, the iTOL server 6.8.2 [32] was utilized for annotation and tree visualization.

## 3. Results

### 3.1. Sequencing results

The sequencing was performed in three separate events. The first event involved the five original samples, the second event included the same samples along with an additional third sequencing batch comprising samples from mosquitoes collected in the localities of Las Lajitas, Santa Fe, and Joaquín V. González. The reads resulting from these three sequencing events were merged into a FastQ file per locality. Subsequently, filtering using BLASTN was conducted, and a FastQ file per locality containing only the viral hits was obtained (Table 3). Top BLAST hit for each read per locality is shown in S3 Table.

The primary source of contamination was from the *Aedes aegypti* transcriptome, posing a significant challenge due to the high presence of EVEs encoded within the *Aedes aegypti* genome. Including reads derived from EVEs could lead to errors or misinterpretations regarding the biological origin of these viral elements, potentially resulting in reference mapping of viruses that are not truly present in the sample. Additionally, *Aedes aegypti* rRNA and bacterial rRNA were prominent sources of contamination; all rRNA-derived reads were filtered out to mitigate this. Finally, contamination from the human genome was also detected, although it was less problematic for the subsequent steps.

After completing all filtering steps, we determined the N50 for each dataset. Despite the capability of nanopore sequencing systems to generate long reads, the N50 for most reads was close to 300 bases in several cases. The only dataset with a higher N50 was from Las Lajitas, with an N50 of 731 (Table 3).

**Table 3:** Reads after basecalling, demultiplexing and filtering against rRNA, Aedes aegypti AaegL5.0 and Homo sapiens genome databases: Total reads in each sample after sequencing and demultiplexing. After using BLASTN against the viral database each read making viral hit was counted.

Sample	Total Reads	Reads making viral hits	Total Reads N50	Reads making viral hits N50
Quilmes—Buenos Aires	1194374	28883	285	280
City of Santa Fe—Santa Fe	655211	2840	262	315
Galpon—Salta	351036	1481	205	334
Las Lajitas—Salta	899831	31660	245	731
Joaquín V. González—Salta	632873	85399	245	309
Unclassified	226466	N/A	N/A	N/A
**Total**	3959791	150263		

### 3.2. Partially and fully assembled virus genomes

The reference mapping using viral hits resulted in partial coverage in some cases and complete coverage of viruses in others (Table 4). One of the viruses with the highest presence in the samples is the Phasi Charoen-like Phasivirus (PCLV), which was found and fully assembled across the three regions where mosquitoes were captured. It was fully assembled with high coverage degree and depth in three cities comprising Quilmes, Santa Fe and Joaquín V. Gonzalez. It was partially assembled in the city of Las Lajitas.

**Fig 2:**
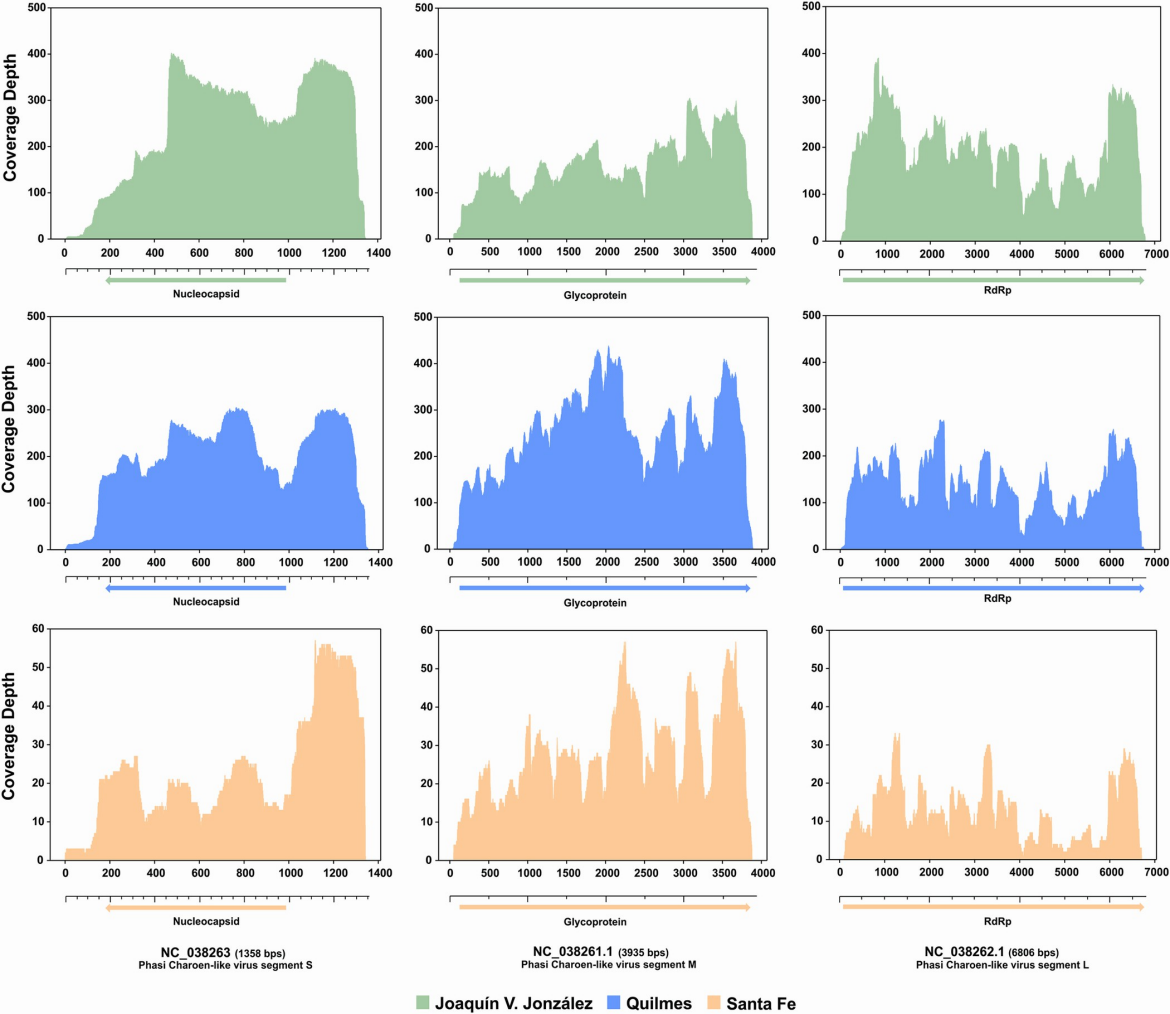
Genome coverage plots of Phasi Charoen-like Phasivirus Segments. Sequencing reads from different samples were mapped on to the reference sequences for S (small, NC_038263.1), M (medium, NC_038261.1) and L (large, NC_038262.1) segments, respectively, retrieved from GenBank. The X-axis indicates genome position, Y axis indicates sequencing depth. Below each graph, the ORFs of each segment are schematically represented. Collection sites are indicated with different colors.

The coverage of reads across the reference genomes in the fully sequenced viruses was observed. The three segments of PCLV were mapped in the three regions (Salta, Buenos Aires, and Santa Fe), each showing >97% sequence coverage (Table 4). BAM coverage plots show that the sequence close to the 5’ and 3’ ends are the least covered, but the rest of the genome are fully sequenced and mapped against the reference sequence (Fig 2).

**Fig 3:**
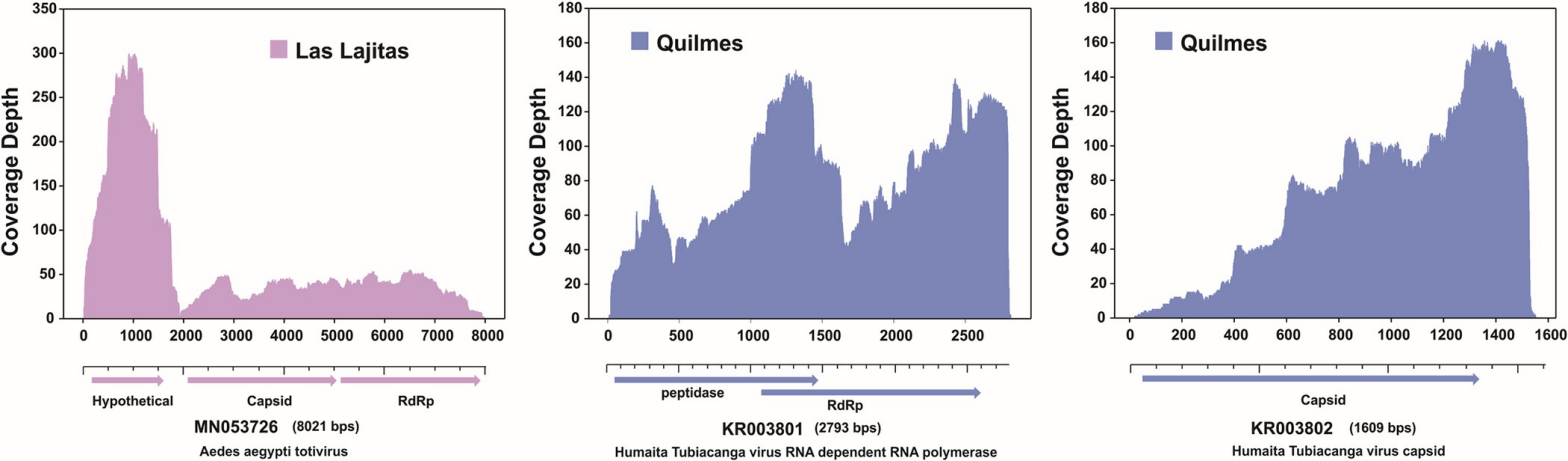
Genome coverage plots of Humaita Tubiacanga Virus genomic segments and Aedes aegypti Totivirus. Sequencing reads from different samples were mapped on to the reference sequences for Humaita Tubiacanga Virus–RdRp (MN053809.1), Humaita Tubiacanga Virus–Capsid (MN053810.1), retrieved from GenBank. The X-axis indicates genome position, Y axis indicates sequencing depth. Below each graph, the ORFs of each segment are schematically represented. Collection sites are indicated with different colors.

Among other viruses detected, we have fully mapped and assembled Humaita Tubiacanga Virus (HTV) and Aedes aegypti totivirus (AaTV) (Fig 3). Both genomic segments of HTV were fully sequenced in the samples of Quilmes and Santa Fe. Capsid and RdRp segments showed coverage above 97%, but the assembly from Santa Fe has low mean depth (<18). In the case of AaTV, it was mapped and assembled in the sample belonging to Las Lajitas with a sequence coverage above 99%. AaTV was partially detected in samples from Quilmes, Santa Fe, Galpón and Joaquín V. González city. Only one sample showed the presence of partial genomic regions of Dengue virus serotype 2, from Las Lajitas in the province of Salta.

In order to determine if there is a high grade of SNP’s or collapsed cuasi species, for the fully covered genomes (above 97% of coverage and >12 mean depth) we evaluated the base frequency at each position in the BAM files generated after the reference sequence alignment. The base frequency distribution at each position is above 0.8 in most cases. While there are positions where the frequency falls below this value, the trend indicates that, position by position, the sequences show a high percentage of conservation. Several SNPs are observed compared to the reference sequence, exhibiting variability relative to it. Despite this, within the alignment, the frequency of SNPs is low (S1 Document).

**Table 4:** Mean depth and percentage of coverage of fully and partially assembled viral genomes.

Sample Location	Reference name	Reference Accession number	Consensus length	Reads	per. coverage	mean depth	mean base Q
Quilmes—Buenos Aires	Humaita Tubiacanga Virus—RdRp	MN053809.1	2686	586	100	83.1	20.7
	Humaita Tubiacanga Virus—Capsid	MN053810.1	1436	260	100	75.5	20.7
	Aedes aegypti totivirus	MT913596.1	2897	17	36.10	0.55	20.7
	Phasi Charoen-like Phasivirus Segment S	NC_038263.1	1352	923	99.56	195.19	21.9
	Phasi Charoen-like Phasivirus Segment M	NC_038261.1	3860	2948	98.1	243.2	21.5
	Phasi Charoen-like Phasivirus Segment L	NC_038262.1	6754	3094	99.2	136.45	21.4
City of Santa Fe—Santa Fe	Humaita Tubiacanga Virus—RdRp	MN053809.1	2686	146	100	17.15	20.3
	Humaita Tubiacanga Virus—Capsid	MN053810.1	1404	30	97.77	7.78	20.7
	Aedes aegypti totivirus	MT913596.1	888	3	11.07	0.15	18.7
	Phasi Charoen-like Phasivirus Segment S	NC_038263.1	1343	124	98.9	22.7	22.6
	Phasi Charoen-like Phasivirus Segment M	NC_038261.1	3833	372	97.4	27.3	21.1
	Phasi Charoen-like Phasivirus Segment L	NC_038262.1	6608	296	97.1	12.04	21.4
Galpón—Salta	Aedes aegypti totivirus	MT913596.1	4746	44	59.15	1.88	21.9
Las Lajitas—Salta	Humaita Tubiacanga Virus—RdRp	MN053809.1	2651	12	98.69	2.48	20.7
	Humaita Tubiacanga Virus—Capsid	MN053810.1	699	3	48.68	0.53	18.2
	Aedes aegypti Totivirus	MN053726.1	7978	883	99.44	69.49	21.43
	Phasi Charoen-like Phasivirus Segment S	NC_038263.1	945	15	69.58	2.30	19.8
	Phasi Charoen-like Phasivirus Segment M	NC_038261.1	3378	34	85.84	2.97	19.5
	Phasi Charoen-like Phasivirus Segment L	NC_038262.1	3752	22	55.12	0.98	20.2
	Dengue Virus serotype 2	OR025676.1	2140	2	20.55	0.20	19.8
Joaquín V. González—Salta	Humaita Tubiacanga Virus—RdRp	MN053809.1	511	1	19.02	0.19	20.6
	Aedes aegypti Totivirus	MN053726.1	7573	195	94.39	8.28	21.6
	Phasi Charoen-like Phasivirus Segment S	NC_038263.1	1344	953	99.0	239.6	21.8
	Phasi Charoen-like Phasivirus Segment M	NC_038261.1	3844	1772	97.7	156.66	21.2
	Phasi Charoen-like Phasivirus Segment L	NC_038262.1	6785	3620	99.7	189.56	21.5

### 3.3. Phasi Charoen-like phasivirus

Phasi Charoen-like phasivirus (PCLV) is one of the most prevalent viruses in the *Aedes aegypti* virome. It belongs to the phlebovirus genus of the *Phenuiviridae* family. PCLV, in conjunction with Humaita Tubiacanga virus (HTV), has been shown to enhance arbovirus replication in mosquitoes and further the transmission of clinically important arboviruses such as Zika virus and Dengue virus [11].

**Fig 4:**
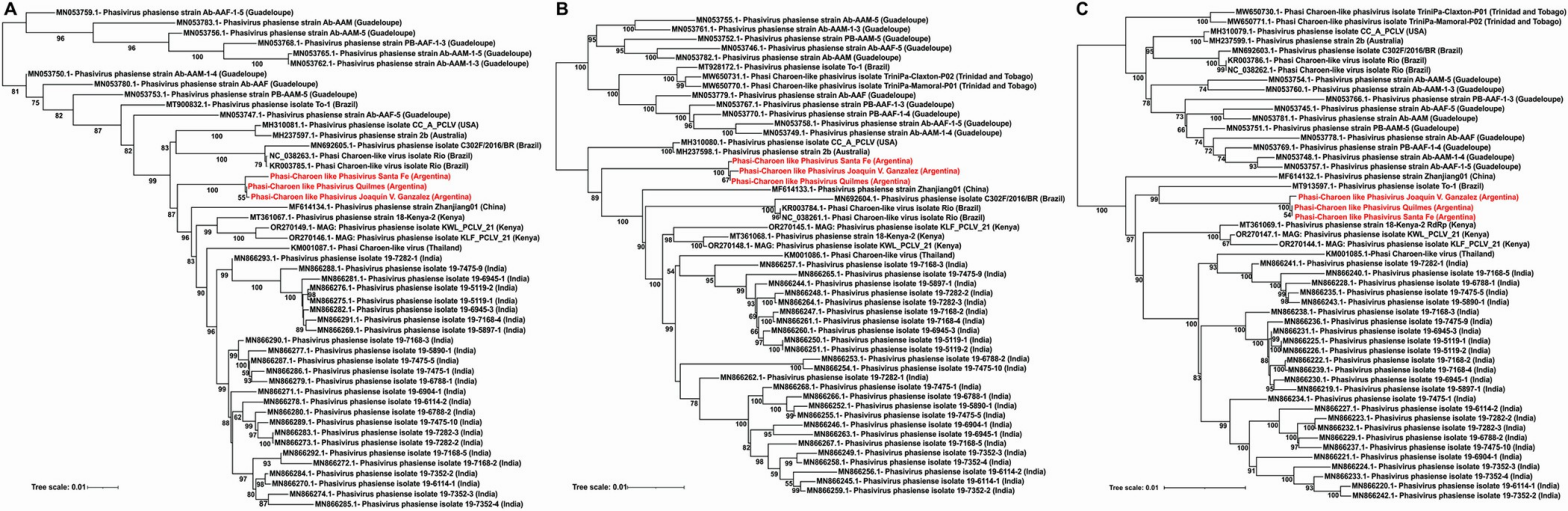
Phylogeny inference Phasi Charoen-like phasivirus (PCLV) based on its three genomic segments (S, M and L). Maximum-likelihood tree based on the nucleotide sequences of full-length S (Panel A), M (Panel B) and L (Panel C) genomic segments of PCVL virus. Prototypes and sequences obtained in this work were used. Each virus is identified by the genome ID (Genbank), the name of the virus with its corresponding strain or isolate, and the country of origin in parentheses. The three Argentine PCLV isolations, identified in this study, are in red. Bootstrap values greater than 50 are indicated. Maximum-likelihood phylogenetic tree was inferred using the GTR+F+R2 model and 1000 replicates in every case.

PCLV has a negative-stranded ssRNA genome consisting of three segments called L, M, and S. Segment S has a size of 1.3 kb and encodes the nucleocapsid gene; segment M has a length of 3.8 kb, encoding the glycoprotein gene; and segment L has a size of 6.8 kb, encoding the RNA-dependent RNA polymerase gene [33]. We detected, reference-mapped, and assembled all three segments of this virus in three distinct regions of Argentina: Quilmes in Buenos Aires province, the City of Santa Fe in Santa Fe province, and Joaquin V. González in Salta Province. Phylogenetic analysis was conducted using the full nucleotide sequence of the three genomic segments, revealing that the three PCLV isolates obtained in Argentina are grouped in a clade with maximum consistency values (100 bootstrapping), being even separated from the isolates obtained in Brazil (S4 Table and Fig 4). For the three segments (S, M and L), an identity percentage greater than 98.0% was observed among the three Argentine isolates (98.0–99.8); while, when compared with the rest of the reported sequences, the identity values varied depending on the segment analyzed. In the case of the L segment, RdRp gene, identity values between 95.2–96.9 were mostly obtained; and only with four isolates from Brazil and Kenya (MT913597.1, OR270147.1, MT361069.1, OR270144.1) values greater than 97% identity (97.0–97.3) were found (See S7 Table for identity matrix). For the M segment, the percentage of identity with the rest of the reported sequences was 92.9–96.6; while for the S segment the greatest variability was observed, with identity percentages from 43.3 to 96.5. These results allow us to hypothesize that the Argentine isolates belong to an isolated clade, exhibiting a very close evolutionary relationship between themselves.

### 3.4. Humaita-Tubiacanga virus

**Fig 5:**
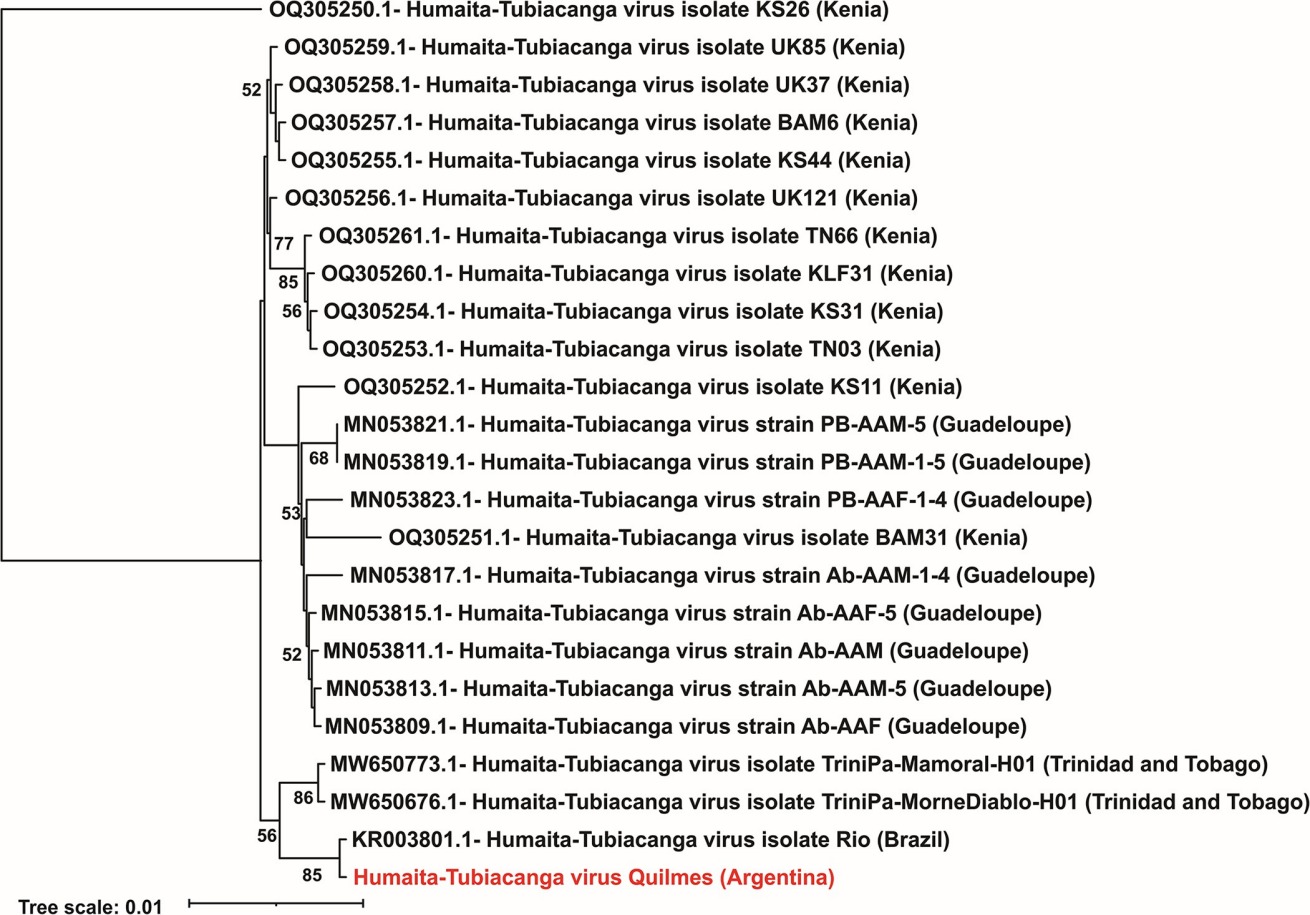
Phylogeny inference of Humaita-Tubiacanga virus based on RNA-dependent RNA polymerase protein. Maximum-likelihood tree based on full-length RNA-dependent RNA polymerase (RdRp) aminoacidic sequences from Humaita-Tubiacanga virus prototypes and the genome obtained in this work. Each virus is identified by the genome ID (Genbank), the name of the virus with its corresponding strain or isolate, and the country of origin in parentheses. The Argentinian HTV isolate, identified in this study, is in red. Bootstrap values greater than 50 are indicated. Maximum-likelihood phylogenetic tree was inferred using the HIVb (HIV between-patient matrix HIV-Bm) + F + I model and 1000 replicates.

HTV is a newly detected virus that has not yet been taxonomically classified. Its genome consists of two segments: one segment of 2.8 Kb encodes an RNA-dependent RNA polymerase, and the other of 1.5 Kb encodes the capsid protein. In this study, we detected and assembled the full genome of HTV from mosquito samples collected in Quilmes city, Buenos Aires province. The 1533 nt fragment encodes a 433 amino acid protein that presents a 99% similarity with the capsid protein (AKP18619) of the first Brazilian HTV isolate (KR003801) [34]. The 2808 nt fragment encodes two main open reading frames. An open reading frame encoding a 460 amino acid protein that presents a 98% similarity with a protein of the Brazilian HTV (AKP18618), annotated as "replicase" in this genome but as a hypothetical protein in the other GenBank genomes. The functional characterization with InterProScan server (https://www.ebi.ac.uk/interpro/) allows us to assume that this hypothetical protein is a peptidase (Peptidase S1, PA clan, IPR009003). The other ORF encodes a 505 amino acid protein that presents a 98% similarity with the RdRp from Kenya isolate (WDR17589). Phylogenetic analysis of RdRp amino acid sequence, obtained by *in silico* translation of the genomic segments, shows that Quilmes isolated sequence grouped in a separate clade with the first sequence of HTV isolated in Brazil (S5 Table and Fig 5). These two sequences have a similarity percentage of 98.42%, while the identity percentage varies between 95.45 and 98.39 with the rest of sequences. However, it should be noted that all HTV sequences show a high conservation (similarity values ​​of 95.45–100, excluding the outgroup).

### 3.5. Aedes aegypti totivirus

**Fig 6:**
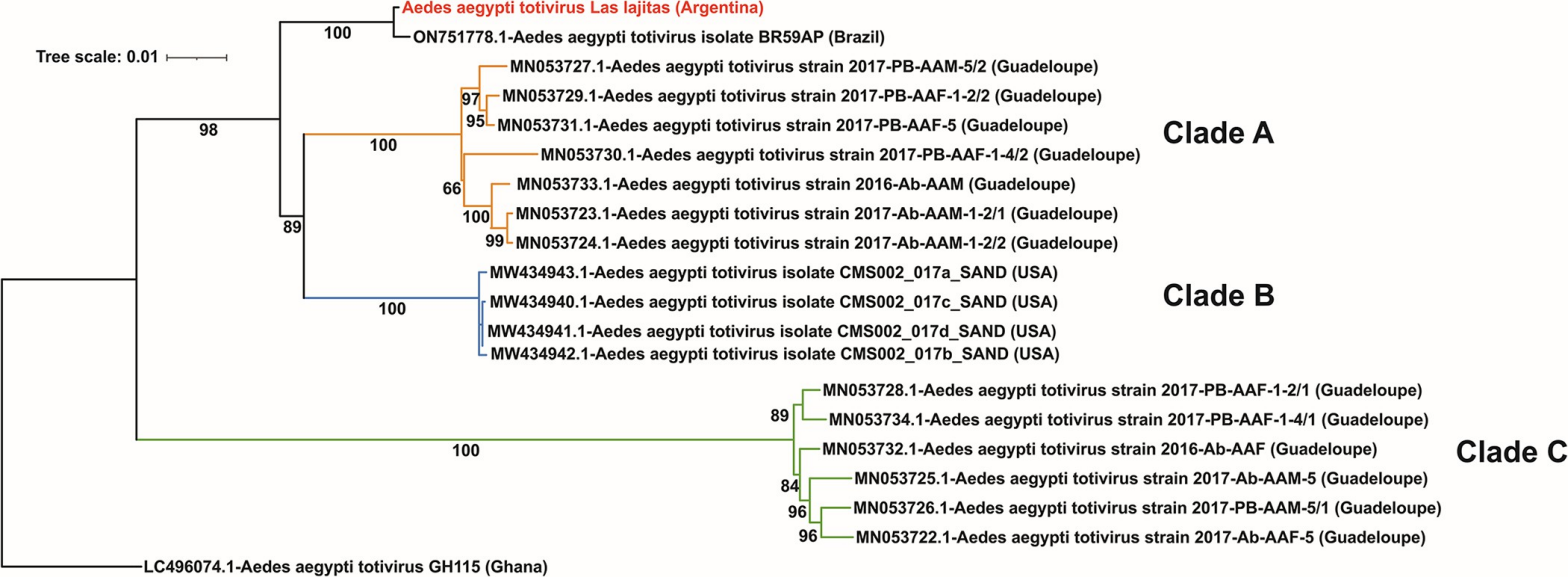
Phylogeny inference of Totivirus based on RNA-dependent RNA polymerase gene. Maximum-likelihood tree based on full-length RNA-dependent RNA polymerase (RdRp) nucleotide sequences from Totivirus prototypes and the genome obtained in this work. Each virus is identified by the genome ID (Genbank), the name of the virus with its corresponding strain or isolate, and the country of origin in parentheses. The Argentinian Totivirus isolate, identified in this study, are in red. The branches of each main phylogenetic clades (A, B, C) are colored. Bootstrap values greater than 50 are indicated. Maximum-likelihood phylogenetic tree was inferred using the General Time Reversible (GTR) +F + G4 distribution model, Gamma shape alpha of 0.1301 and 1000 replicates.

Aedes aegypti totivirus (AaTV) is an unclassified virus belonging to the Totiviridae family. This virus has a dsRNA genome of 7.9 Kbp which encodes for a capsid protein and an overlapped ORF encoding for an RNA-dependent RNA polymerase [35]. In this study, we have recovered the full genome of AaTV (7976 nucleotides) from mosquito samples captured in Las Lajitas, Province of Salta. Using the nucleotide sequence of the RNA-dependent RNA polymerase, we constructed a maximum likelihood phylogenetic tree comparing our sequence with isolates from Brazil, USA, Guadeloupe Island, and Ghana (S6 Table). The tree shows three distinctive clades, previously identified [35], formed by the Guadeloupe Island and USA isolates (denominated A, B, and C); and a fourth clade composed of the isolates from Brazil and from Las Lajitas obtained in this work (Fig 6). Las Lajitas and Brazilian isolates make up a new clade (100 bootstrap), clearly separated from clades A and B. The percentage of identity between these two sequences is 98.5%; while identity percentage varies between 89.5–94.8 with the rest of sequences. In this case, it is also important to mention that when the complete identity matrix is analyzed, identity percentages greater than 98% only occur when comparing sequences from the same clade. This supports the proposal that the South American isolates would belong to another new clade. Furthermore, in this new reported genome the 3 characteristic ORFs were detected; 915-amino acid protein has a similarity percentage of 97% with an RdRp of Aedes aegypti totivirus (QEM39153.1),. The two other ORFs encoding proteins of 906 and 471 amino acids, and presenting 97% similarity with the capsid protein (QEM39152) and 96% similarity with a hypothetical protein (QEM39144) of Aedes aegypti totivirus, respectively. No similarity was detected with the amino acid sequence from Brazil because it was not annotated in the database. According to these evolutionary analyses, it could be inferred that the South American isolates come from an ancestor that clearly differs from those from which clades A and B arise. However, to confirm these proposals, more analysis of genomic comparisons should be carried out.

## 4. Discussion

This work aimed to characterize the virome of wild mosquitoes from Argentina using nanopore-based NGS technology. For this, adult mosquitoes and eggs were collected during the year 2022–2023 in different regions of Argentina (Salta, Buenos Aires and Santa Fe). We implement a scheme to process the samples and select the fraction containing viral particles and budding vesicles. To identify the RNA virome from mosquitoes, a methodology based on SISPA was implemented. Subsequently, libraries compatible with nanopore NGS technology were built. Finally, the data obtained was analyzed bioinformatically using a pipeline designed *ad-hoc*, based on genomic identification and assembly using reference databases.

The bioinformatic analysis comprised thorough filtering steps including BLASTN against several ribosomal gene databases. This was necessary because several reads were found with homology to a single virus-specific genomic region and to some ribosomal genomic sequences. This indicates that the procedure used to extract and purify viral RNA must be more exhaustive when depleting circulating RNA. Subsequently, the data was filtered with the human genome database, since various reads with homology with human genes were found. Also a final filtering step was conducted against the *Aedes aegypti* genome assembly AaegL5.0. This was an essential step in order to avoid the inclusion of reads derived from the endogenous viral elements integrated in the mosquito genome.

The implementation of our workflow, combining SISPA methodology with Nanopore sequencing and a bioinformatics pipeline designed for its analysis, allowed us to identify the presence of several ISVs in mosquitoes from different areas of Argentina. From the samples collected in northern Argentina, specifically in Galpón, Las Lajitas and Joaquín V. González in Salta, we proceeded to work only with the isolates from the latter two locations due to the higher number of reads. In Joaquín V. González isolate the complete genome sequence of PCLV was obtained, while in Las Lajitas we obtained partial genomic segments with more than 50% coverage in the 3 segments. Likewise, AaTV was completely sequenced in Las Lajitas isolate, almost completely in the Joaquín V. González isolate (93% coverage, 8.28 mean depth) and detected in low proportion in samples from El Galpón (59% coverage). In addition, in Joaquín V. González isolate we obtained a sequence corresponding to a fragment of the HTV genome. Phylogenetic and sequence identity analyses allowed us to determine that “Las Lajitas” AaTV isolate is more related to the Brazilian isolate, forming a new South American isolate clade separated from the rest. Similarly, PCLV Joaquín V. González isolate appears to be more ancestrally related to other Argentine isolates than other countries. Furthermore, the characterization of the PCLV L segment allowed us to directly link the Argentine samples with one of the Brazilian isolates. Finally, it is important to mention that a genomic region of Dengue virus serotype 2 was partially detected in Las Lajitas isolate, a location with reported Dengue outbreaks. Particularly in Salta Province DENV 2 was reported as the primary circulating serotype during the 1st and 14th epidemiological week of 2023.

On the other hand, of the sample collected from Quilmes we detected the full genomic sequence of HTV and PCLV, while we obtained a partial genomic sequence of AaTV (32%). The PCLV genome has similar characteristics to those of the genome obtained in Joaquín V. González, and forms a clade with the other Argentine samples closely related to one Brazilian isolate. Remarkably, Quilmes isolate was the only one in which the complete HTV genome was recovered. The two genomic segments presented a higher percentage of identity with the segments of the only HTV isolated in Brazil, and the phylogenetic analysis of the RdRp amino acid sequence links both isolates through a node. Additionally, the samples from Santa Fe also contained the full genomic sequences of PCLV. The presence of this virus in Santa Fe isolate, corresponding to adult mosquitoes reared from eggs collected in 2022, suggests a possible vertical transmission mechanism; as these adult specimens were raised in the laboratory from eggs collected using ovitraps. Vertical transmission of several ISVs, such as Cell fusing agent virus (CFAV) and PCLV, has already been documented [36–37]. AaTV was detected in the Santa Fe isolate but obtaining only partial genome sequences.

It is important to highlight that the methodology presented in this work is practical and suitable for understanding the mosquito virome. Therefore, it could be used as a tool for characterizing the viral diversity present in mosquitoes, not only for detecting viruses that have a high impact on human and animal health, but also for detecting ISV’s; which would be interesting given their ability to interact with arbovirus transmission.

## Supporting information

S1 Document Base frequency distribution plot per complete assembled viral genome. (DOCX)

S1 Table Date accession and full links to databases. (XLSX)

S2 Table Filtered Reads. (XLSX)

S3 Table BLAST top Hits of Filtered reads. (XLSX)

S4 Table Accession number of Phasi charoen-like Phasivirus sequences involved in phylogeny inference. (XLSX)

S5 Table Accession number of Humaita Tubiacanga viral sequences involved in phylogeny inference. (XLSX)

S6 Table Accession number of Aedes aegypti Totivirus viral sequences involved in phylogeny inference. (XLSX)

S7 Table Percentage of identity matrix. (XLSX)

## References

[1] Finlay C. J. (2012). The Mosquito Hypothetically Considered as the Transmitting Agent of Yellow Fever. MEDICC review, 14(1), 56–59. doi: 10.37757/MR2012V14.N1.10 34503309

[2] Weaver S. C., Charlier C., Vasilakis N., & Lecuit M. (2018). Zika, Chikungunya, and Other Emerging Vector-Borne Viral Diseases. Annual review of medicine, 69, 395–408. doi: 10.1146/annurev-med-050715-105122 28846489 PMC6343128

[3] Chala B., & Hamde F. (2021). Emerging and Re-emerging Vector-Borne Infectious Diseases and the Challenges for Control: A Review. Frontiers in public health, 9, 715759. doi: 10.3389/fpubh.2021.715759 34676194 PMC8524040

[4] Kraemer M. U. G., Reiner R. C., Jr, Brady O. J., Messina J. P., Gilbert M., Pigott D. M., et al. (2019). Past and future spread of the arbovirus vectors Aedes aegypti and Aedes albopictus. Nature microbiology, 4(5), 854–863. doi: 10.1038/s41564-019-0376-y 30833735 PMC6522366

[5] Robert M. A., Stewart-Ibarra A. M., & Estallo E. L. (2020). Climate change and viral emergence: evidence from Aedes-borne arboviruses. Current opinion in virology, 40, 41–47. doi: 10.1016/j.coviro.2020.05.001 32569752 PMC7305058

[6] Duarte M. A., Campos F. S., Araújo Neto O. F., Silva L. A., Silva A. B., Aguiar T. C., et al. (2022). Identification of potential new mosquito-associated viruses of adult Aedes aegypti mosquitoes from Tocantins state, Brazil. Brazilian journal of microbiology: [publication of the Brazilian Society for Microbiology], 53(1), 51–62. doi: 10.1007/s42770-021-00632-x 34727360 PMC8882499

[7] Souza-Neto J. A., Powell J. R., & Bonizzoni M. (2019). Aedes aegypti vector competence studies: A review. Infection, genetics and evolution: journal of molecular epidemiology and evolutionary genetics in infectious diseases, 67, 191–209. doi: 10.1016/j.meegid.2018.11.009 30465912 PMC8135908

[8] Agboli E., Leggewie M., Altinli M., & Schnettler E. (2019). Mosquito-Specific Viruses-Transmission and Interaction. Viruses, 11(9), 873. doi: 10.3390/v11090873 31533367 PMC6784079

[9] Hobson-Peters J., Yam A. W., Lu J. W., Setoh Y. X., May F. J., Kurucz N., et al. (2013). A new insect-specific flavivirus from northern Australia suppresses replication of West Nile virus and Murray Valley encephalitis virus in co-infected mosquito cells. PloS one, 8(2), e56534. doi: 10.1371/journal.pone.0056534 23460804 PMC3584062

[10] Romo H., Kenney J. L., Blitvich B. J., & Brault A. C. (2018). Restriction of Zika virus infection and transmission in Aedes aegypti mediated by an insect-specific flavivirus. Emerging microbes & infections, 7(1), 181. 10.1038/s41426-018-0180-4 30429457 PMC6235874

[11] Olmo R. P., Todjro Y. M. H., Aguiar E. R. G. R., de Almeida J. P. P., Ferreira F. V., Armache J. N., et al. (2023). Mosquito vector competence for dengue is modulated by insect-specific viruses. Nature microbiology, 8(1), 135–149. doi: 10.1038/s41564-022-01289-4 36604511

[12] de Almeida J. P., Aguiar E. R., Armache J. N., Olmo R. P., & Marques J. T. (2021). The virome of vector mosquitoes. Current opinion in virology, 49, 7–12. doi: 10.1016/j.coviro.2021.04.002 33991759

[13] Kumar A., Murthy S., & Kapoor A. (2017). Evolution of selective-sequencing approaches for virus discovery and virome analysis. Virus research, 239, 172–179. doi: 10.1016/j.virusres.2017.06.005 28583442 PMC5819613

[14] Zakrzewski M., Rašić G., Darbro J., Krause L., Poo Y. S., Filipović I., et al. (2018). Mapping the virome in wild-caught Aedes aegypti from Cairns and Bangkok. Scientific reports, 8(1), 4690. doi: 10.1038/s41598-018-22945-y 29549363 PMC5856816

[15] Xiao P., Li C., Zhang Y., Han J., Guo X., Xie L., et al. (2018). Metagenomic Sequencing From Mosquitoes in China Reveals a Variety of Insect and Human Viruses. Frontiers in cellular and infection microbiology, 8, 364. doi: 10.3389/fcimb.2018.00364 30406041 PMC6202873

[16] Thannesberger J., Rascovan N., Eisenmann A., Klymiuk I., Zittra C., Fuehrer H. P., et al. (2020). Highly Sensitive Virome Characterization of Aedes aegypti and Culex pipiens Complex from Central Europe and the Caribbean Reveals Potential for Interspecies Viral Transmission. Pathogens (Basel, Switzerland), 9(9), 686. doi: 10.3390/pathogens9090686 32839419 PMC7559857

[17] Thannesberger J., Rascovan N., Eisenmann A., Klymiuk I., Zittra C., Fuehrer H. P., et al. (2021). Viral metagenomics reveals the presence of novel Zika virus variants in Aedes mosquitoes from Barbados. Parasites & vectors, 14(1), 343. doi: 10.1186/s13071-021-04840-0 34187544 PMC8244189

[18] Langat S. K., Kerich G., Cinkovich S., Johnson J., Ambale J., Yalwala S., et al. (2023). Genome sequences of Phasi Charoen-like phasivirus and Fako virus from Aedes aegypti mosquitoes collected in coastal Kenya. Microbiology resource announcements, 12(11), e0067823. doi: 10.1128/MRA.00678-23 37846988 PMC10652973

[19] Reyes G. R., & Kim J. P. (1991). Sequence-independent, single-primer amplification (SISPA) of complex DNA populations. Molecular and cellular probes, 5(6), 473–481. doi: 10.1016/s0890-8508(05)80020-9 1664049

[20] Chrzastek K., Lee D. H., Smith D., Sharma P., Suarez D. L., Pantin-Jackwood M., & Kapczynski D. R. (2017). Use of Sequence-Independent, Single-Primer-Amplification (SISPA) for rapid detection, identification, and characterization of avian RNA viruses. Virology, 509, 159–166. doi: 10.1016/j.virol.2017.06.019 28646651 PMC7111618

[21] Chrzastek K., Tennakoon C., Bialy D., Freimanis G., Flannery J., & Shelton H. (2022). A random priming amplification method for whole genome sequencing of SARS-CoV-2 virus. BMC genomics, 23(1), 406. doi: 10.1186/s12864-022-08563-z 35644636 PMC9148844

[22] Marcacci M., De Luca E., Zaccaria G., Di Tommaso M., Mangone I., Aste G., et al. (2016). Genome characterization of feline morbillivirus from Italy. Journal of virological methods, 234, 160–163. doi: 10.1016/j.jviromet.2016.05.002 27155238 PMC7172958

[23] Schulz A., Sadeghi B., Stoek F., King J., Fischer K., Pohlmann A., et al. (2022). Whole-Genome Sequencing of Six Neglected Arboviruses Circulating in Africa Using Sequence-Independent Single Primer Amplification (SISPA) and MinION Nanopore Technologies. Pathogens (Basel, Switzerland), 11(12), 1502. doi: 10.3390/pathogens11121502 36558837 PMC9781818

[24] Brinkmann A., Uddin S., Krause E., Surtees R., Dinçer E., Kar S., et al. (2021). Utility of a Sequence-Independent, Single-Primer-Amplification (SISPA) and Nanopore Sequencing Approach for Detection and Characterization of Tick-Borne Viral Pathogens. Viruses, 13(2), 203. doi: 10.3390/v13020203 33572847 PMC7911436

[25] Toh X., Wang Y., Rajapakse M. P., Lee B., Songkasupa T., Suwankitwat N., et al. (2022). Use of nanopore sequencing to characterize african horse sickness virus (AHSV) from the African horse sickness outbreak in thailand in 2020. Transboundary and emerging diseases, 69(3), 1010–1019. doi: 10.1111/tbed.14056 33682298

[26] Crispell G., Williams K., Zielinski E., Iwami A., Homas Z., & Thomas K. (2022). Method comparison for Japanese encephalitis virus detection in samples collected from the Indo-Pacific region. Frontiers in public health, 10, 1051754. doi: 10.3389/fpubh.2022.1051754 36504937 PMC9730272

[27] Altschul S. F., Gish W., Miller W., Myers E. W., & Lipman D. J. (1990). Basic local alignment search tool. Journal of molecular biology, 215(3), 403–410. doi: 10.1016/S0022-2836(05)80360-2 2231712

[28] Li H. (2018). Minimap2: pairwise alignment for nucleotide sequences. Bioinformatics (Oxford, England), 34(18), 3094–3100. doi: 10.1093/bioinformatics/bty191 29750242 PMC6137996

[29] Sievers F., & Higgins D. G. (2021). The Clustal Omega Multiple Alignment Package. Methods in molecular biology (Clifton, N.J.), 2231, 3–16. doi: 10.1007/978-1-0716-1036-7_1 33289883

[30] Danecek P., Bonfield J. K., Liddle J., Marshall J., Ohan V., Pollard M. O., et al. (2021). Twelve years of SAMtools and BCFtools. GigaScience, 10(2), giab008. doi: 10.1093/gigascience/giab008 33590861 PMC7931819

[31] Minh B. Q., Hahn M. W., & Lanfear R. (2020). New Methods to Calculate Concordance Factors for Phylogenomic Datasets. Molecular biology and evolution, 37(9), 2727–2733. doi: 10.1093/molbev/msaa106 32365179 PMC7475031

[32] Letunic I., & Bork P. (2021). Interactive Tree Of Life (iTOL) v5: an online tool for phylogenetic tree display and annotation. Nucleic acids research, 49(W1), W293–W296. doi: 10.1093/nar/gkab301 33885785 PMC8265157

[33] Chandler J. A., Thongsripong P., Green A., Kittayapong P., Wilcox B. A., Schroth G. P., et al. (2014). Metagenomic shotgun sequencing of a Bunyavirus in wild-caught Aedes aegypti from Thailand informs the evolutionary and genomic history of the Phleboviruses. Virology, 464–465, 312–319. doi: 10.1016/j.virol.2014.06.036 25108381 PMC4157124

[34] Aguiar E. R., Olmo R. P., Paro S., Ferreira F. V., de Faria I. J., Todjro Y. M., et al. (2015). Sequence-independent characterization of viruses based on the pattern of viral small RNAs produced by the host. Nucleic acids research, 43(13), 6191–6206. doi: 10.1093/nar/gkv587 26040701 PMC4513865

[35] Leal É., Ribeiro E. S. D., Monteiro F. J. C., Marques J. P., Dos Santos Mendes D., Morais V. S., et al. (2023). Aedes aegypti Totivirus identified in mosquitoes in the Brazilian Amazon region. Virus genes, 59(1), 167–172. doi: 10.1007/s11262-022-01955-z 36394716

[36] Logan R. A. E., Quek S., Muthoni J. N., von Eicken A., Brettell L. E., Anderson E. R., et al. (2022). Vertical and Horizontal Transmission of Cell Fusing Agent Virus in Aedes aegypti. Applied and environmental microbiology, 88(18), e0106222. doi: 10.1128/aem.01062-22 36036577 PMC9499017

[37] Zhang X., Huang S., Jin T., Lin P., Huang Y., Wu C., et al. (2018). Discovery and high prevalence of Phasi Charoen-like virus in field-captured Aedes aegypti in South China. Virology, 523, 35–40. doi: 10.1016/j.virol.2018.07.021 30077072

