# Supplementary material for: Insect-specific RNA viruses detection in Field-Caught Aedes aegypti mosquitoes from Argentina using NGS technology
Source: PLoS Negl Trop Dis. 2025 Jan 10;19(1):e0012792. doi: 10.1371/journal.pntd.0012792 (PMC11756794; doi:10.1371/journal.pntd.0012792)
Supplement: S1 Document — (DOCX) [file pntd.0012792.s001.docx]

**Base Frequency distribution plots**

Barcode 1 – Quilmes, Buenos Aires Province

Humaita Tubiacanga Virus – Capsid


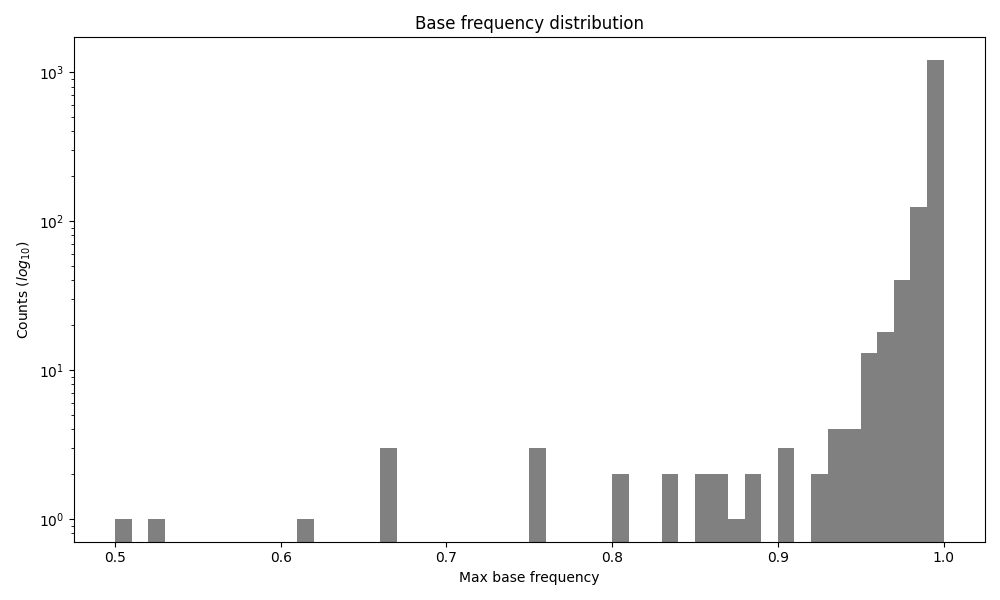


Humaita Tubiacanga Virus – RdRp


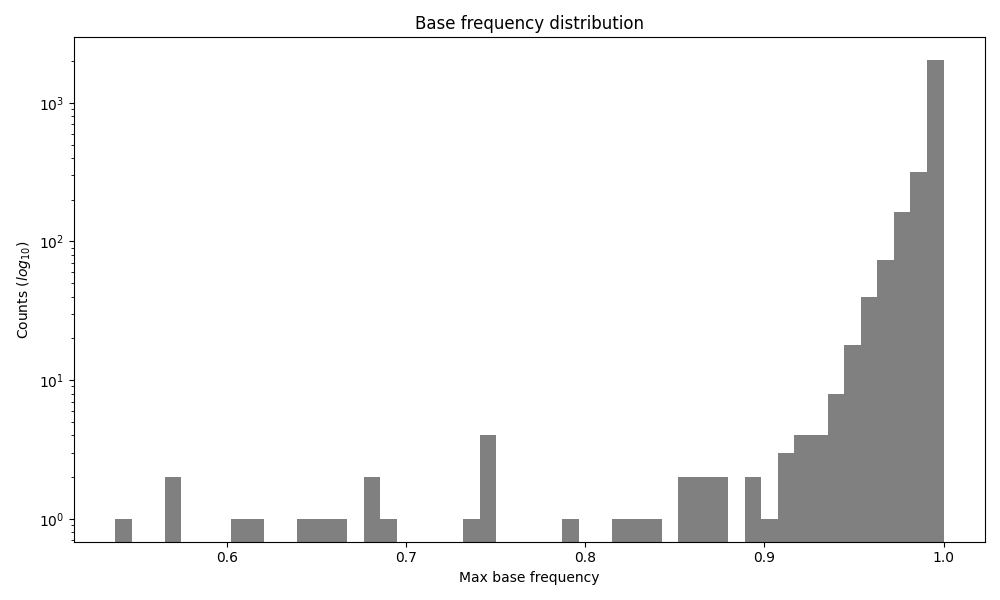


Phasi-Charoen Like Phasivirus – Segment L


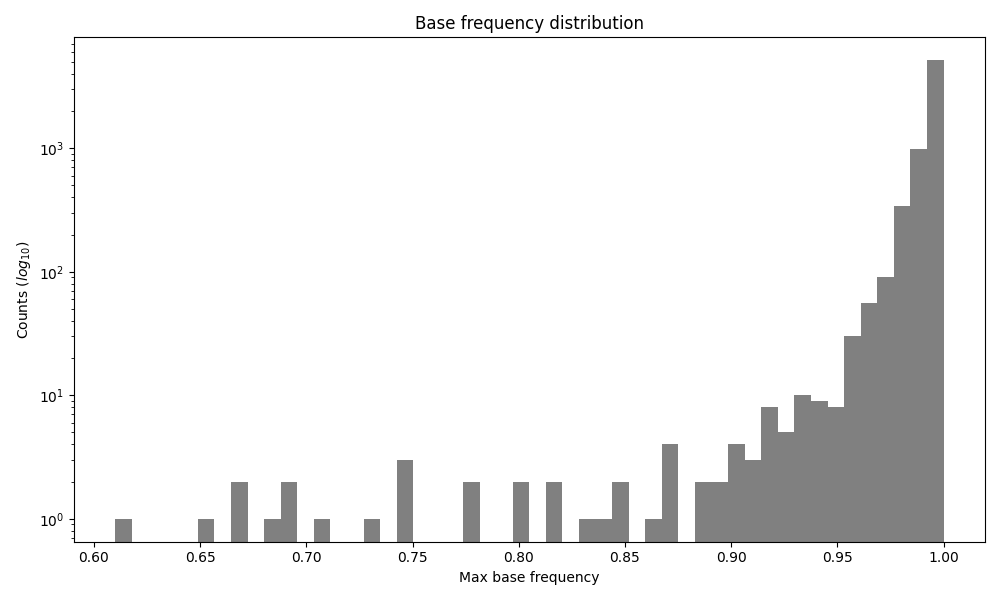


Phasi-Charoen Like Phasivirus – Segment M


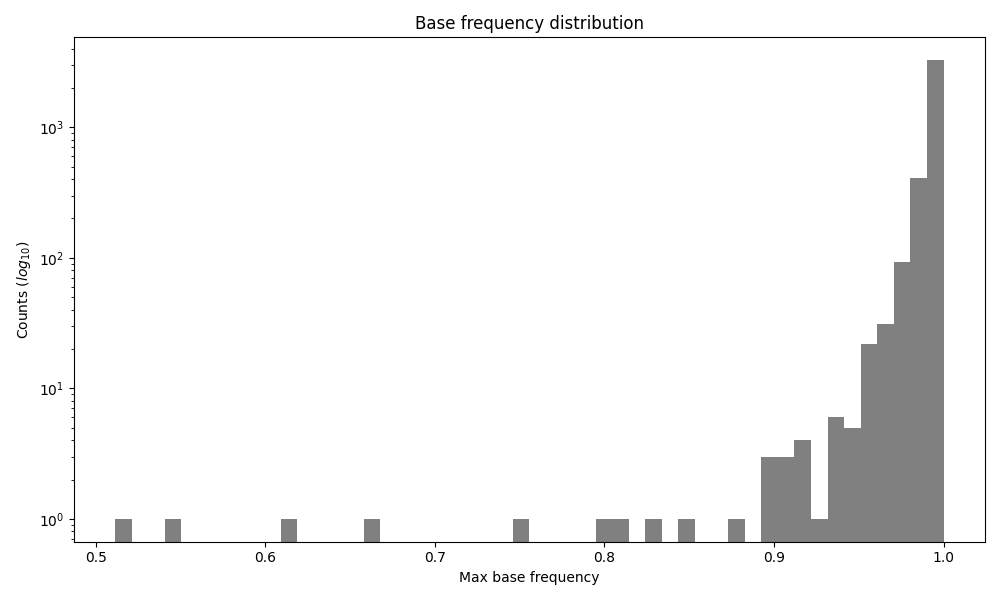


Phasi-Charoen Like Phasivirus – Segment S


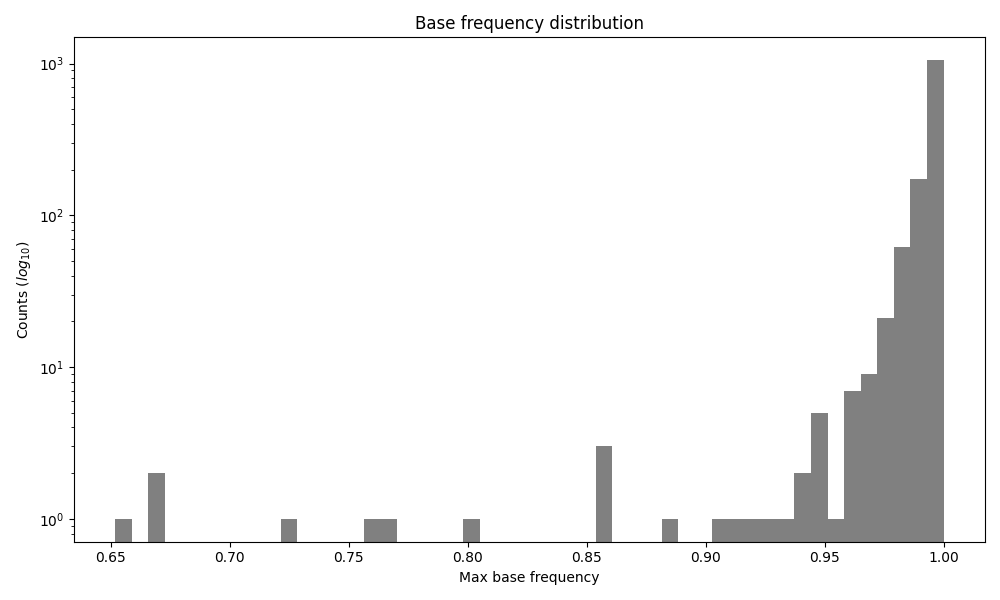


Barcode 2 – Santa Fe, Santa Fe Province

Phasi-Charoen Like Phasivirus – Segment L


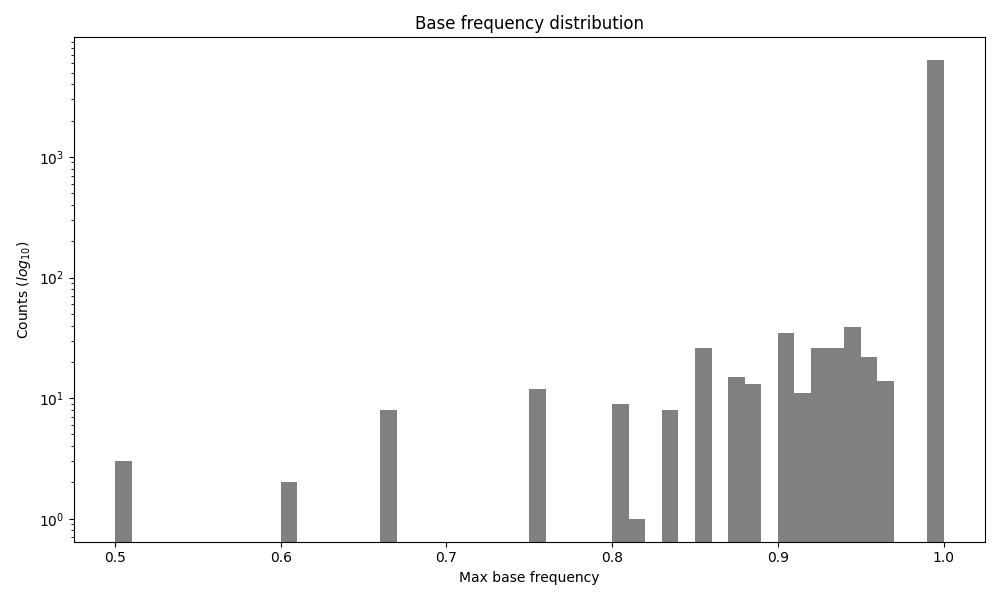


Phasi-Charoen Like Phasivirus – Segment M


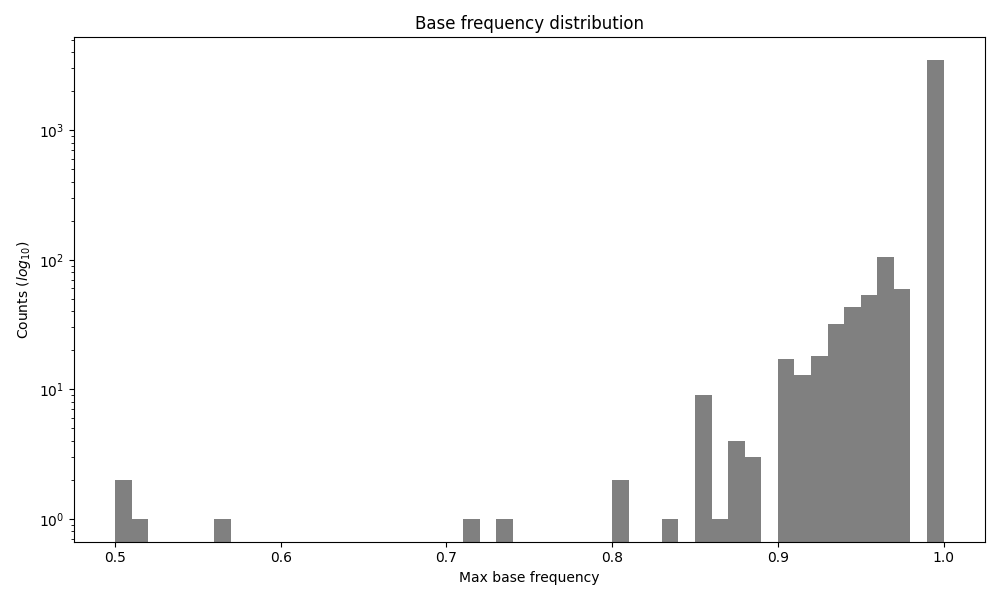


Phasi-Charoen Like Phasivirus – Segment S


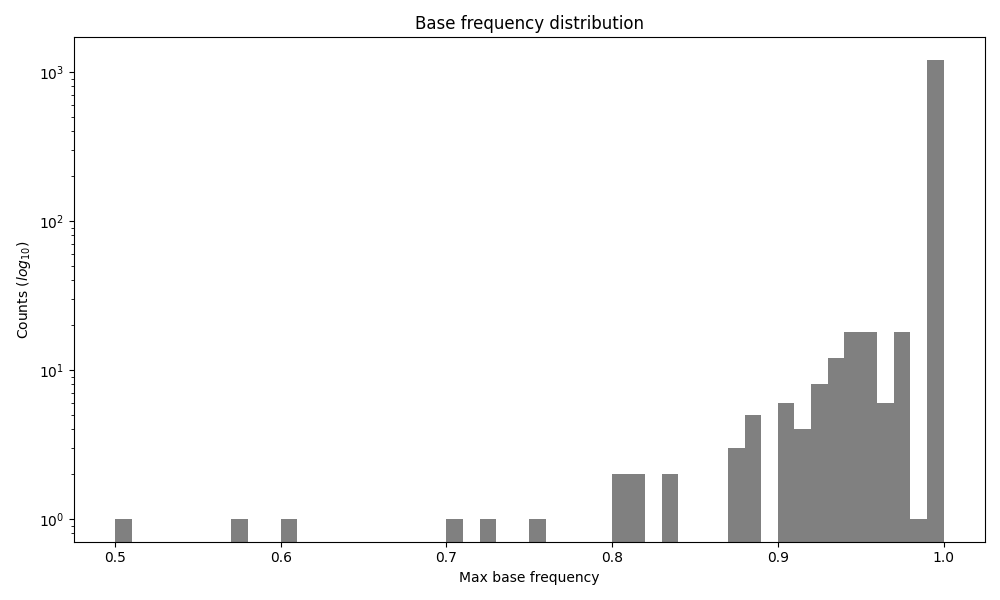


Barcode 4 – Las Lajitas, Salta Province

Aedes Aegypti Totivirus


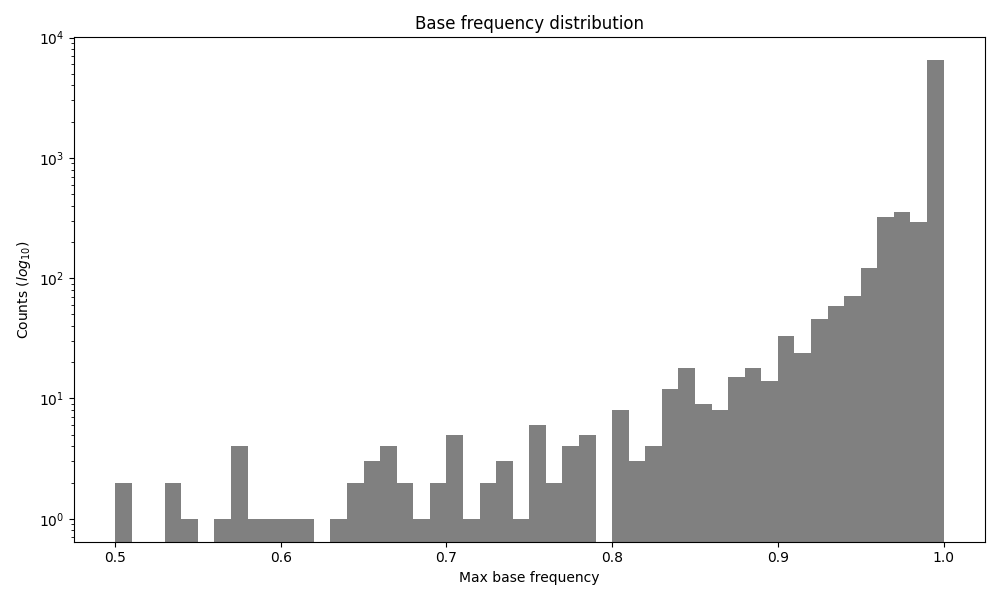


Barcode 5 – Joaquin V. Gonzalez, Salta Province

Phasi-Charoen Like Phasivirus – Segment L


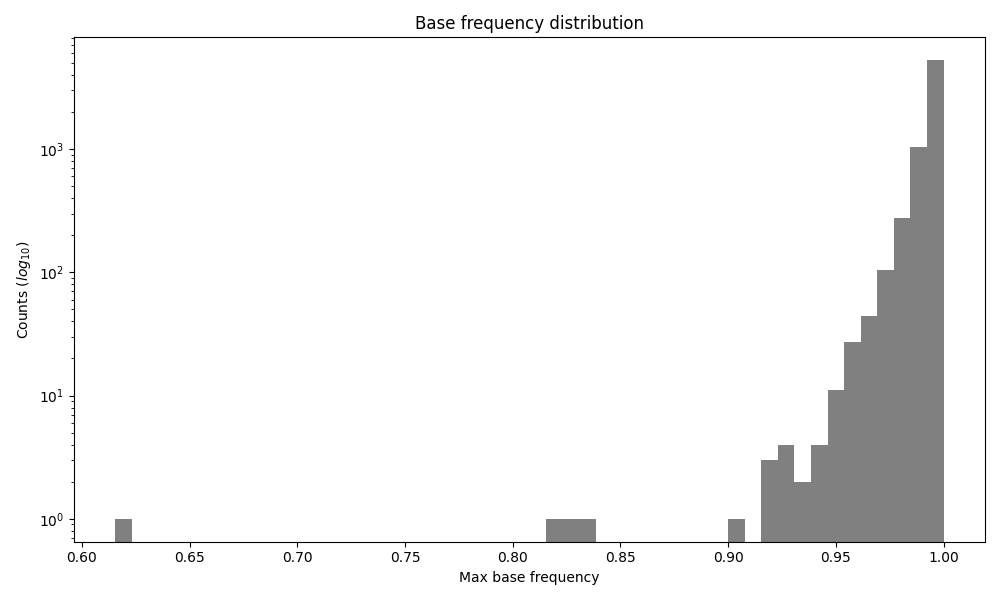


Phasi-Charoen Like Phasivirus – Segment M


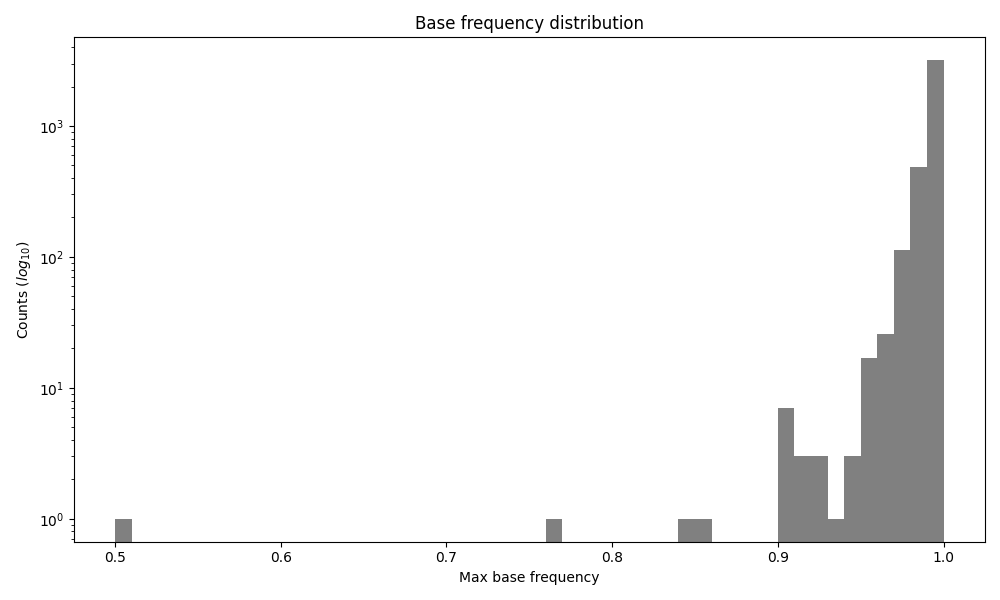


Phasi-Charoen Like Phasivirus – Segment S


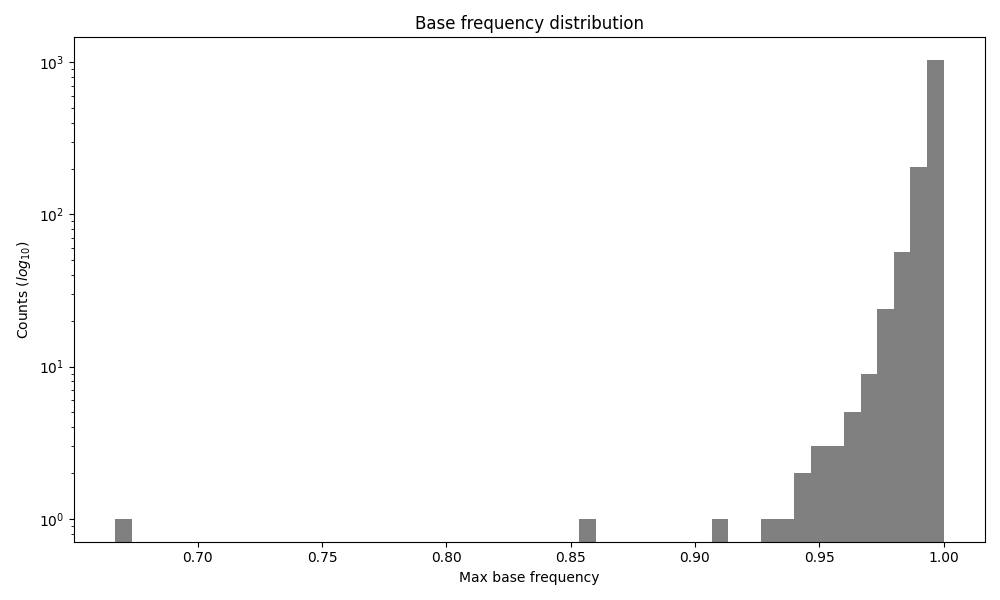


Quantile compiled Table

|  | **q10** | **q5** | **q1** |
| --- | --- | --- | --- |
| **barcode1_humaita_capsid** | 0.98361 | 0.972429 | 0.859155 |
| **barcode1_humaita_pol** | 0.97826 | 0.96774 | 0.89655 |
| **barcode1_phasi_charoen_L** | 0.98571 | 0.97959 | 0.9438139 |
| **barcode1_phasi_charoen_M** | 0.987432 | 0.98131 | 0.9546962 |
| **barcode1_phasi_charoen_S** | 0.987504 | 0.980334 | 0.9261804 |
| **barcode1_to_virus 2** | 1 | 0.94444 | 0.8 |
| **barcode2_to_virus 2** | 1 | 1 | 0.7303595 |
| **barcode2_phasi_charoen_S** | 1 | 0.95238 | 0.875 |
| **barcode2_phasi_charoen_M** | 1 | 0.95455 | 0.90909 |
| **barcode2_phasi_charoen_L** | 1 | 1 | 0.85714 |
| **barcode4_aedes_totivirus** | 0.98113 | 0.94737 | 0.8239652 |
| **barcode5_phasi_charoen_S** | 0.987751 | 0.982558 | 0.9628028 |
| **barcode5_phasi_charoen_L** | 0.98718 | 0.98182 | 0.96429 |
| **barcode5_phasi_charoen_M** | 0.98558 | 0.980458 | 0.959748 |
